# EpiPro, a Novel, Synthetic, Activity-Regulated Promoter That Targets Hyperactive Neurons in Epilepsy for Gene Therapy Applications

**DOI:** 10.3390/ijms241914467

**Published:** 2023-09-23

**Authors:** Cassidy T. Burke, Iuliia Vitko, Justyna Straub, Elsa O. Nylund, Agnieszka Gawda, Kathryn Blair, Kyle A. Sullivan, Lara Ergun, Matteo Ottolini, Manoj K. Patel, Edward Perez-Reyes

**Affiliations:** 1 Department of Pharmacology, University of Virginia, Charlottesville, VA 22908, USA; 2 Computational and Predictive Biology, Oak Ridge National Laboratory, Oak Ridge, TN 37830, USA; 3 Department of Anesthesiology, University of Virginia, Charlottesville, VA 22908, USAmkp5u@virginia.edu (M.K.P.); 4 UVA Brain Institute, University of Virginia, Charlottesville, VA 22908, USA

**Keywords:** epilepsy, promoter, transcription factor, AAV, hippocampus, dentate gyrus

## Abstract

Epileptogenesis is characterized by intrinsic changes in neuronal firing, resulting in hyperactive neurons and the subsequent generation of seizure activity. These alterations are accompanied by changes in gene transcription networks, first with the activation of early-immediate genes and later with the long-term activation of genes involved in memory. Our objective was to engineer a promoter containing binding sites for activity-dependent transcription factors upregulated in chronic epilepsy (EpiPro) and validate it in multiple rodent models of epilepsy. First, we assessed the activity dependence of EpiPro: initial electrophysiology studies found that EpiPro-driven GFP expression was associated with increased firing rates when compared with unlabeled neurons, and the assessment of EpiPro-driven GFP expression revealed that GFP expression was increased ~150× after status epilepticus. Following this, we compared EpiPro-driven GFP expression in two rodent models of epilepsy, rat lithium/pilocarpine and mouse electrical kindling. In rodents with chronic epilepsy, GFP expression was increased in most neurons, but particularly in dentate granule cells, providing in vivo evidence to support the “breakdown of the dentate gate” hypothesis of limbic epileptogenesis. Finally, we assessed the time course of EpiPro activation and found that it was rapidly induced after seizures, with inactivation following over weeks, confirming EpiPro’s potential utility as a gene therapy driver for epilepsy.

## 1. Introduction

Temporal lobe epilepsy (TLE) is a common subtype of focal epilepsy [1]. It is also difficult to control with drugs, such that one-third of TLE patients are considered drug-resistant [2]. An effective treatment for TLE is surgical resection of the affected temporal lobe, but this procedure can result in adverse cognitive effects, such as the impairment of verbal memory and reasoning [3]. The lack of safe and effective treatment options currently available to TLE patients presents a need for a novel therapeutic approach. One approach tested in animal models has been to use gene therapies based on adeno-associated viral (AAV) delivery [4]. An ideal therapy for TLE would restore the balance of excitation and inhibition in hippocampal circuits, preventing seizures from originating there and minimizing off-target effects. To this end, we describe the development of a promoter with selectivity for epileptic neurons (EpiPro) and characterize the hippocampal circuits it can act on after intraparenchymal AAV injection. Other approaches that have been used to target overly active neurons are the use of the cFos promoter [5] and NPAS4 response elements in the so-called Robust Activity Marking (RAM) promoter [6].

**Figure 1:**
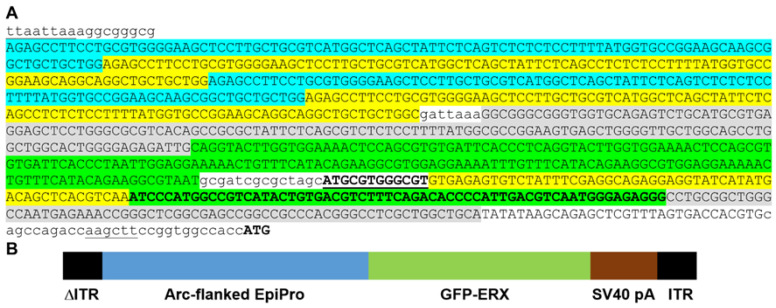
Nucleotide sequence of the Arc-flanked EpiPro promoter and experimental design. (**A**), The promoter begins with 5 copies of the distal Arc elements described by Bito and coworkers [12,19]. Each element includes CREB, MEF2, and SRF response elements. To reduce inadvertent recombination due to repetitive sequences, we alternated the nucleotide sequence between those found in rat (blue), mouse (yellow), or human Arc distal promoter elements (gray). The core EpiPro promoter begins with 5 copies of the NFAT-AP1 response element (green; TRANSFAC record M00302). The first 2 copies are from the Bgt1 gene, while the next 3 copies are from the IL2 gene [27]. The IL2 response elements were modified from the luciferase reporter vector pGL4.30 (Promega Corporation, Madison, WI, USA). The same sequences have been validated in vivo [10]. This was followed by AsiSI and NheI restriction sites and a single EGR RE (bold, underlined, TRANSFAC record M00243). Next are the 3 calcium response elements from the mouse BDNF gene (yellow; CaRE1, CaRE2, and CaRE3/CRE [13]). The core EpiPro promoter ends with 2 copies of the cyclic AMP RE (bold green, TRANSFAC record M00178) modified from Promega’s luciferase reporter vector pGL4.29 (sequence from Vip and Per2 genes). This is followed by the human proximal Arc element (gray, [12]) and a synthetic initiation region that includes the CMV minimal promoter [28] as well as a consensus USF1-binding site at the transcription start site (CACGTG, TRANSFAC record M00217). Shown in lowercase letters are the restriction enzymes that flank the promoter (underlined) and linker sequences. The starting ATG codon for the GFP protein is noted in bold. (**B**), Diagram of the scAAV targeting vector containing the promoter, eGFP, and the polyadenylation sequence from SV40. The eGFP was codon-optimized, and an ER export signal was added to enhance trafficking (FCYENE, [29]). The targeting vector contains the deletion in the 5′ internal terminal repeat (ΔITR) that leads to the packaging of double-stranded, self-complementary DNA [15].

Pioneering work by Loeb’s group characterized the transcriptional profile of human epileptic tissue, finding an upregulation of cAMP Response Element-Binding Protein (CREB)-regulated genes [7,8]. Many of these genes are also upregulated after chronic stimulation of neurons in vitro [9]. Importantly, the transcription factor response elements in the promoters of these genes have been mapped, allowing us to design a synthetic promoter composed of these DNA sequences. The core EpiPro promoter consists of the following response elements upregulated in epilepsy: nuclear factor of activated T-cells (NFAT); early growth response protein (EGR); calcium response elements (CaRE) from brain-derived neurotrophic factor (BDNF); and cAMP response elements for CREB (Figure 1A). Each of these elements alone can drive the expression of reporter genes such as luciferase [10,11,12,13]. Since these elements may be active in distinct subsets of neurons, they were combined to target the majority of epileptic neurons. Other key features of our approach included the following: (1) we used the rh10 serotype of the AAV coat protein, which transduces hippocampal neurons at high efficiency over a wide area [14]; (2) we used a self-complementary AAV design that enhances expression [15], allowing for transient in vitro assays [16]; (3) we used convective delivery of AAVs [17]; and (4) the EpiPro promoters drove the expression of a codon-optimized GFP containing an ER export signal that improves the trafficking of optogenetic tools in neurons ([18], Figure 1B). In contrast, most studies of activity-regulated promoters use destabilized reporters to study transient changes in neuronal activity [19]. For example, fusion of the ornithine decarboxylase degradation domain to GFP reduces its half-life from 26 to 5 hrs [20]. The ER-export-enhanced GFP was chosen to allow the quantitation of native fluorescence as a readout of long-term changes in neuronal activity.

After confirming the activity dependence of EpiPro with initial experiments, we sought to characterize the neurons where EpiPro is active in control and epileptic rats and map their projections throughout the brain. We used the well-characterized Li/pilocarpine rat model, where a single injection of pilocarpine induces a long-lasting seizure (*status epilepticus*, SE), leading to the development of spontaneous limbic seizures [21]. A key finding was the massive upregulation of EpiPro expression in dentate granule cells from epileptic animals. This result provides strong support for the “breakdown of the dentate gate” theory of limbic seizure initiation [22,23], which posits that the loss of GABAergic inhibition disrupts the filtering function of the dentate gyrus, leading to the hyperactivation of dentate granule cell firing [24]. We found similar results using mice and seizures triggered by either electrical kindling [25] or an intraperitoneal injection of kainic acid. Finally, we assessed the time course of the activation and decay of EpiPro by utilizing a mouse model that we developed in which seizures self-remit [26]. We found that EpiPro activates within one day after a seizure and that this activity is maintained for two weeks, with basal activity maintained between seizures. This favorable time course of action validates the potential use of EpiPro to drive gene therapies for epilepsy.

## 2. Results

### 2.1. Validation of the Activity Dependence of EpiPro in Rats

The initial validation of EpiPro promoters was performed in vitro using dual-luciferase reporter assays after transfection into mammalian cell lines. These studies confirmed that EpiPro was regulated by cAMP and its transcriptional regulator, CREB. We then tested two versions of EpiPro after the infection of cultured hippocampal neurons using recombinant self-complementary AAV (scAAV): EpiPro (EPRGFP) and EpiPro flanked by Arc promoter elements (ADGFP, sequence in Figure 1A). These Arc elements included five copies of the distal promoter and a single copy of the proximal regulatory elements [19]. These studies illustrated the activity dependence of EpiPro promoters and showed that the distal Arc elements increased GFP expression approximately 5-fold.

**Figure 2:**
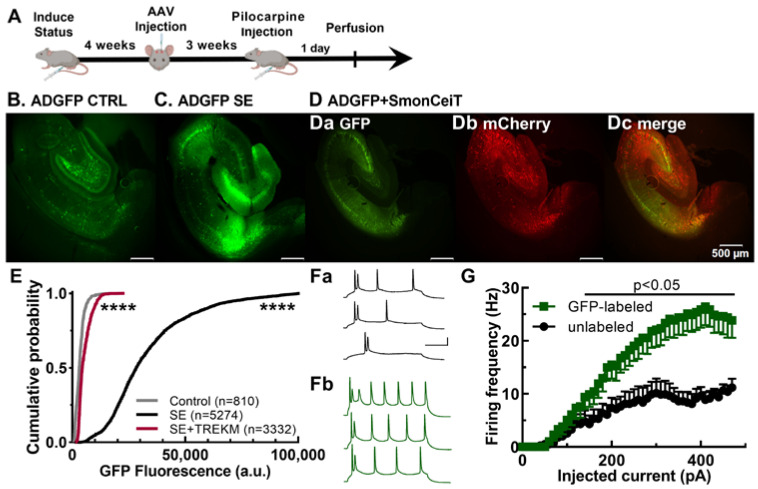
Validation of EpiPro as an activity-dependent promoter. (**A**), Timeline of experimental procedure. (**B**–**E**), In vivo studies after intraparenchymal injection of scADGFP alone or with an AAV encoding both TREK-M and mCherry (SmonCeiT). The control group was only injected with AAV, while the SE groups were injected with Li/pilocarpine. (**B**–**D**), Representative 4× images of an animal from each group: (**B**) control of scADGFP alone; (**C**) scADGFP + SE; (**D**) scADGFP + SE + SmonCeiT. Brightness of the green fluorescence was adjusted to allow comparison relative to SE (**C**); therefore, control is enhanced 4-fold (**B**), and SE +TREK-M is enhanced 2-fold (**Da**). Also shown from the SE + TREK-M experiment are the mCherry fluorescent signal (**Db**) and the merge with the GFP signal (**Dc**). (**E**), Cumulative probability plot of the GFP fluorescence in DGCs from the same 3 groups. Statistical analysis by one-way ANOVA (Kruskal–Wallis test) followed by Dunn’s test; all 3 groups were significantly different from each other (*p* < 0.0001, ****; N is the number of DGCs shown). (**F**,**G**), Current-clamp recordings of subicular neurons in brain slices from 2 control rats injected with scADGFP. Neurons were patched using the whole-cell configuration, and then their firing frequencies were evaluated as a function of depolarizing current injections. Recordings were made on unlabeled (**Fa**) and GFP-expressing cells (**Fb**) identified using epifluorescence. Representative traces obtained after 170, 279, and 450 pA for each condition. Scale bar represents 100 ms and 25 mV. (**G**), Plot of the average firing frequency as a function of injected current (*n* = 13 neurons/group). Statistical analysis used a mixed-effects model followed by Tukey’s multiple-comparison test.

We next studied EpiPro activation in vivo using a double stimulus that should produce maximal activation with a chemoconvulsant-triggered seizure in chronic epileptic rats (Figure 2A, Table 1). We used the Li/pilocarpine procedure, which reliably induces status epilepticus (SE) with low mortality [30]. Rats that survive (~75%) develop epilepsy but often show clustering of seizures with seizure-free intervals [31]. Therefore, we triggered a second bout of SE with Li/pilocarpine and perfused the rats one day later to assess EpiPro-driven GFP expression. To reduce neuronal death, we stopped motor seizures 10 min after they began with diazepam and then fixed their brains 20 h later. Results from animals injected with scADGFP but not treated with Li/pilocarpine are labeled “Control”. GFP expression was clearly observed in controls, as detailed below. Status epilepticus triggered a 10-fold increase in both the number of GFP-labeled neurons and their GFP fluorescence (Figure 2B–D). We estimate the dynamic range of scADGFP to be 150-fold, based on the sum of GFP fluorescence. In this experiment, we also cotransfected scADGFP with a second AAV that directs the expression of TREK-M (SmonCeiT), a mutant leak K^+^ channel [32]. TREK-M was selected because it reliably reduces the neuronal firing of cultured neurons and, after AAV delivery, reduces the severity and duration of SE [30], as well as spontaneous seizures [32]. Accordingly, TREK-M inhibited GFP expression from scADGFP by 80% (Figure 2C,D).

We also validated the activity dependence of EpiPro using current-clamp recordings from brain slices. These studies focused on the firing of subicular neurons in controls, comparing GFP-labeled neurons to adjacent unlabeled neurons. Action potential firing was induced by depolarizing current injections using methods that we have described previously [33]. Representative traces and average input–output curves are shown in Figure 2F,G, respectively. On average, GFP-labeled neurons fired at significantly higher frequencies (2.5-fold) after an injection of more than 140 pA depolarizing current. Notably, there was no significant difference between GFP-labeled and non-labeled cells in terms of either resting membrane potential (labeled, −64.2 ± 0.6 mV, *n* = 13; unlabeled, −63.7 ± 0.3 mV, *n* = 13, *p* = 0.4 unpaired *t*-test) or input resistance (labeled, 130 ± 12 MΩ, *n* = 13; unlabeled, 137 ± 12 MΩ, *n* = 13, *p* = 0.7 unpaired *t*-test). Taken together, these validation studies establish EpiPro as a marker for highly active neurons.

### 2.2. Experiments Using Epileptic Rats with Spontaneous Seizures

We next studied GFP expression from EpiPro in epileptic animals in which the AAV was either injected before the Li/pilocarpine treatment to induce SE or weeks after treatment when spontaneous seizures were observed (Table 1). The pattern of EpiPro-driven GFP expression was similar between the two groups, so the data were pooled. Their similarity indicates that epilepsy-induced changes in AAV tropism did not affect our results [25]. Motor seizures were monitored using video (frequency ~1/day), and then rats were perfused 1–4 days after their last seizure (Table 1). Following video confirmation of spontaneous seizures, rats were perfused, and their brains were fixed in PFA. Ten horizontal slices from each brain were imaged, uniformly distributed across depths from −4 to −8.5 mm below bregma. To reduce bias, the analysis was performed by researchers blinded to the experimental condition, and we averaged the count of GFP-labeled cells/slice across all slices with GFP-labeled neurons.

### 2.3. Dentate Granule Cells and Their Mossy Fiber Projections

**Figure 3:**
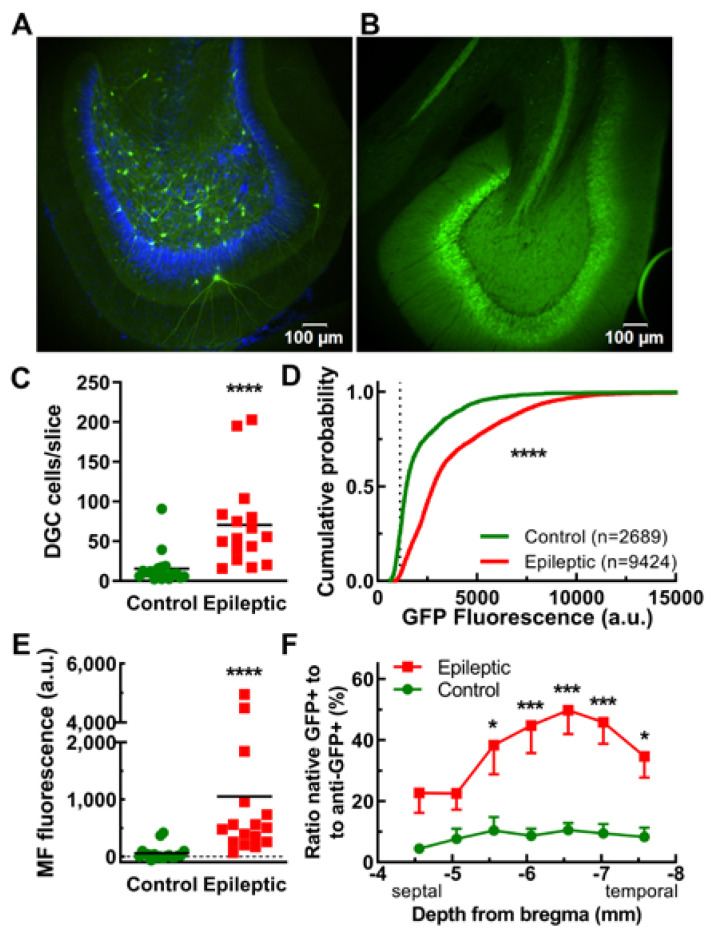
EpiPro-driven expression of GFP in dentate granule cells (DGCs) of control and epileptic rats. Representative images illustrating the lack of DGC labeling in control (**A**) and robust DGC labeling in epileptic animals (**B**). Images shown at the same relative brightness. (**C**), Average number of DGCs counted in each slice (line at mean). Statistical significance was calculated using the Mann–Whitney test (****, *p* < 0.0001; control, *n* = 18; and epileptic, *n* = 16 hemispheres). (**D**), Cumulative probability plot of the mean GFP fluorescence of every neuron analyzed. For counting, we used a “2-fold above background” criterion (dotted line at 1130). Statistical analysis by the Kolmogorov–Smirnov test, where *n* is the number of neurons shown (****, *p* < 0.0001). (**E**), The average GFP fluorescence in the mossy fiber tract (line at mean). Analysis was restricted to areas devoid of labeled CA3 dendrites. Statistical significance was calculated using the Mann–Whitney test (****, *p* < 0.0001; control, *n* = 18; and epileptic, *n* = 16 hemispheres). (**F**), An activity index was calculated by dividing the number of cells counted using the native GFP signal (active) by the number counted in the same slice after anti-GFP antibody staining (total). This activity index is plotted as a function of depth from the top of the brain. Statistical significance was calculated using *t*-tests corrected for multiple comparisons using the Holm–Sidak method (*, *p* < 0.05; ***, *p* < 0.001; *n* = 14 hemispheres for both).

The most striking difference in GFP expression was observed in the dentate granule cell layer (Figure 3A,B). Control animals were observed to have 14 ± 4 labeled DGCs per slice, while epileptic animals had 71 ± 14 DGCs per slice (*n* = 20 and 16 hemispheres, respectively, *p* < 0.0001, Figure 3C; rationale for using 2 hemispheres per animal is detailed in Methods Section 4.9). In addition, the DGCs in epileptic animals had a 2-fold higher GFP intensity than observed in control animals (Figure 3D). The mossy fiber tract was also observed to be significantly brighter in epileptic animals, with an average intensity of 52 ± 27 a.u. in control animals and 1049 ± 374 a.u. in epileptic animals (*n* = 20 and 16 hemispheres, respectively, *p* < 0.0001; Figure 3E). Although the majority of GFP+ cells in the DGC layer appeared to be granule cells, larger cells with distinctive morphology were occasionally observed, such as semilunar granule cells (bottom of Figure 3A image, Table 1). An activity index was calculated by determining the ratio of the count of native GFP-positive neurons to the count of anti-GFP-antibody-stained red neurons (Alexa Fluor^®^ 555). In epileptic animals, the activity index was significantly higher in temporal (−6 to −7 mm depth from bregma) brain slices when compared to septal brain slices, while in control animals, the activity index remained relatively low and constant at each depth (Figure 3F).

### 2.4. CA Neurons

**Figure 4:**
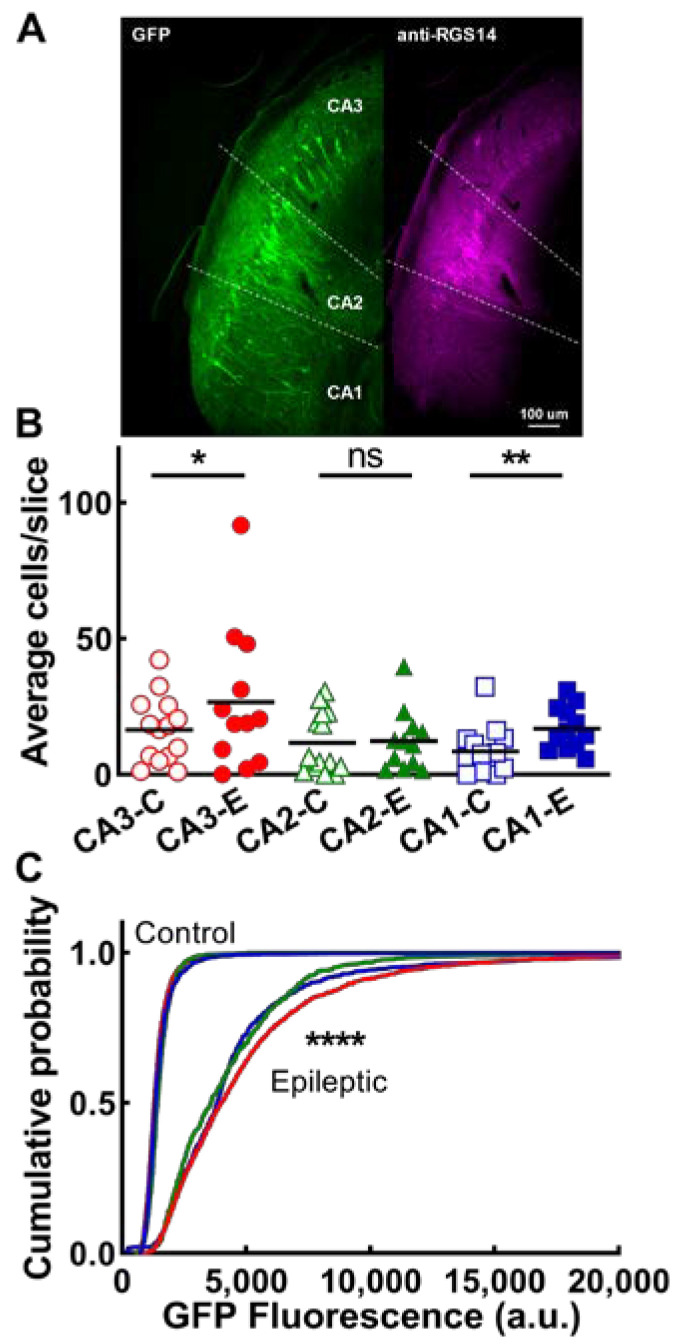
EpiPro-driven expression of GFP in CA neurons of naive and epileptic rats. (**A**), Representative images of GFP expression (GFP) and anti-RGS14 labeling to localize CA2 from CA3 and CA1 (dotted lines). Images taken from a control animal. (**B**), Average number of CA cells observed per slice. Statistical significance was calculated using the Mann–Whitney test (ns, non-significant; *, *p* > 0.05; **, *p* < 0.01; control, *n* = 14; epileptic, *n* = 12 hemispheres). (**C**), Cumulative probability plot of the mean GFP fluorescence of every neuron analyzed. Statistical analysis by the Kolmogorov–Smirnov test. The number of cells used for this analysis were as follows: CA3, control (CA3-C), 2472, epileptic 2284 (CA3-E); CA2, control (CA2-C) 1394, epileptic (CA2-E) 695; and CA1, control (CA1-C) 1039, and epileptic 1096, CA1-E). Data for CA3 in epileptic animals are shown as a red line, CA2 data are shown as a green line, and CA1 data are shown as a blue line. Data for all 3 CA fields in control animals are plotted using the same color scheme as in B, but are indistinguishable. (****, *p* < 0.0001 refers to the statistical difference between epileptic and control for each CA region.

Preliminary experiments with EpiPro-driven GFP expression appeared to show preferential labeling of a cluster of pyramidal neurons at the tip of the hippocampal stratum lucidum: the location of CA2 neurons. To confirm their identity, we stained with a known marker of CA2 neurons, RGS14 ([34]; Figure 4A). These studies confirmed that most of these were indeed CA2 neurons (Figure 4A). However, a subset of CA3 and CA1 neurons were also strongly GFP-labeled in both control and epileptic rats. We then counted GFP-labeled cell bodies in each of the CA layers. On average, epileptic animals showed significantly more GFP+ neurons in CA3 and CA1 than control animals (Figure 4B). In addition, the average intensity of the native GFP signal was significantly higher in epileptic neurons in all three CA fields (Figure 4C). In animals with predominantly unilateral AAV injections into CA3, there was a strong projection to the contralateral hippocampus, extending from the subgranular zone of the dentate to CA1.

### 2.5. Subiculum and Projections

**Figure 5:**
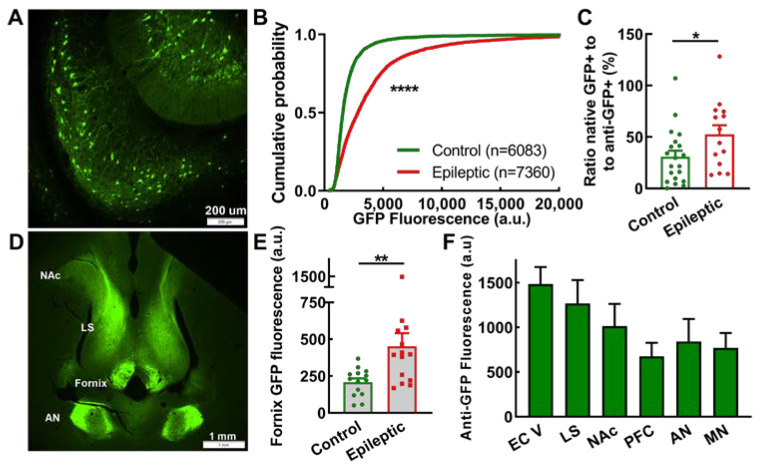
EpiPro-driven expression of GFP in subicular neurons and axonal projections in rats. (**A**), Representative images of GFP expression (GFP) in subicular neurons from a control rat. (**B**), Cumulative probability plot of the mean GFP fluorescence of every neuron analyzed. Statistical analysis by the Kolmogorov–Smirnov test, where N is the number of neurons shown (****, *p* < 0.0001). (**C**), Activity index, as determined by the native to antibody-enhanced counts of GFP-positive neurons. Statistical significance was calculated using the Mann–Whitney test (*, *p* < 0.05; control, *n* = 20; and epileptic, *n* = 14 hemispheres). (**D**), Low-power image of the rostral brain showing axonal projections in the fornix, the anterior nuclear group of the thalamus (AN), the lateral septum (LS), and the nucleus accumbens (NAc). Slice was from an epileptic animal and corresponds to a depth of approximately −6 mm from bregma. (**E**), Average native GFP fluorescence measured in the fornix. Statistical significance was calculated using the Mann–Whitney test (**, *p* < 0.001; control, *n* = 14; and epileptic, *n* = 14 hemispheres). (**F**), Qualitative results of axonal projections observed in epileptic animals to the following brain regions: entorhinal cortex layer V (EC V), lateral septum (LS), nucleus accumbens (NAc), prefrontal cortex (PFC), and anterior (AN) and midline (MN) groups of the thalamus. Values plotted are mean ± SEM from 7 epileptic rats.

The subiculum is a major output structure from the hippocampus proper, sending axons via the fornix to a wide array of brain regions [35,36], and is a common site for spontaneous seizure generation [37]. Therefore, mapping axonal projections from this region can inform how seizures generalize from the hippocampal focus. Subicular neurons were readily labeled by EpiPro (Figure 5A), and as shown in Figure 2F–G, GFP-labeled subicular neurons were capable of firing at higher frequencies. While the number of GFP+ subicular neurons was similar in control and epileptic animals (cells/slice: control, 40 ± 10; epileptic, 42 ± 6; *n* = 20 and 14 hemispheres, respectively; *p* = 0.3 Mann–Whitney test), their average GFP intensity was significantly higher in epileptic animals (Figure 5B). Similarly, the activity index calculated by comparing native to antibody-enhanced GFP counts showed a 2-fold increase in epileptic animals (*p* = 0.04; Figure 5C).

EpiPro drove strong GFP expression in subicular projection neurons, allowing the direct measurement of the fluorescent signal in the fornix. We measured this signal along the descending fornix (approximate depths −4.6 to −8.1 [38]), which is transected in horizontal slices (Figure 5D). GFP expression in the fornix was significantly higher in epileptic compared to control animals (Figure 5E, *p* = 0.007). In contrast, GFP fluorescence in terminal fields was low and, in many animals, could not be distinguished from the background. Therefore, we characterized the antibody-enhanced GFP signal and present semi-quantitative results (Figure 5F). A narrow band of axonal projections was observed to innervate layer 5 of the entorhinal cortex (EC), which terminated at the EC–perirhinal border. Strong projections to the lateral septum were readily observed, even with direct GFP fluorescence, with minimal signal in the medial septum. GFP signals in the prefrontal cortex (corresponding to pre- and infra-limbic structures [38]) and nucleus accumbens were also observed. Projections to the thalamus included the anterior (AN: anterodorsal, anteroventral, and anteromedial) and midline nuclear groups (MN: paraventricular and reuniens). Projections via the fornix continued to the level of the mammillary bodies, which is consistent with their being from subicular neurons [36].

**Table 1:** Key features of rat subjects *.

Animal ID	AAV Injected	Seizure Status	Seizures (sz/Day)	Days after Last Seizure	Days after Inj.	DGC/ Slice	Comments on Unique Features
**Experiment 1: Acute *status epilepticus* triggered by Li/pilocarpine treatment**							
SV234	ADGFP + CSRH1S	control (cage)	nd	--	20	52	
SV235	ADGFP + CSRH1S	control (cage)	nd	--	21	11	SGC, EC
SV236	ADGFP + CSRH1S	control (cage)	nd	--	21	7	SGC, P/E
SV229	ADGFP	status	status	1	23	249	P/E
SV231	ADGFP	status	status	1	23	191	EC, P/E
SV230	ADGFP + SmonCeiT	status	status	1	22	109	GFP low in TREK-M-infected cells
SV232	ADGFP + SmonCeiT	status	status	1	22	65	GFP low in TREK-M-infected cells
**Experiment 2: Chronic TLE, AAV injected before Li/pilocarpine treatment**							
SV273	ADGFP	control (cage)	nd	nd	43	13	SGC
SV249	ADGFP	epileptic	nd	nd	53	43	Extensive HS, post-fixed
SV250	ADGFP	epileptic	nd	nd	57	65	Extensive HS, P/E
SV265	ADGFP	epileptic	nd	1	65	164	Extensive HS, EC
**Experiment 3: Chronic TLE, AAV injected into spontaneously seizing rats weeks after Li/pilocarpine treatment**							
BV311	ADGFP	sz unknown cause	5 (1)	1	25	155	Excluded neither control nor epileptic
BV312	ADGFP	control (video)	0	--	32	11	SGC, OML, P/E
BV315	ADGFP	control (video)	0	--	103	nd	Used for EPhys, not imaged
BV316	ADGFP	control (video)	0	--	104	nd	Used for EPhys, not imaged
BV322	ADGFP	control (video)	0	--	55	21	P/E, OML
BV323	ADGFP	control (video)	0	--	60	5	SGC, EC
BV324	ADGFP	control (video)	0	--	60	7	P/E, OML
BV340	ADGFP	control (video)	0	--	27	2	GFP+ glia at injection site
BV341	ADGFP	control (video)	0	--	27	9	Highly active subicular neurons
BV297	ADGFP	epileptic	5 (0.4)	1	31	48	Extensive HS, P/E, EC
BV302	ADGFP	epileptic	6 (0.5)	3	34	79	Excluded due to >50% atrophy
BV304	ADGFP	single Sz	1 (0.1)	na	54	75	Lowest Sz. freq., no HS, GCD, EC
BV305A	ADGFP	epileptic	2 (0.3)	4	21	nd	Excluded due to >50% atrophy
BV305B	ADGFP	epileptic	6 (0.8)	1	28	40	Extensive HS, P/E, OML, EC
BV306B	ADGFP	epileptic	5 (1.0)	1	24	40	Extensive HS, EC
BV307	ADGFP	epileptic	89 (11)	1	27	88	Extensive HS, P/E, OML, EC
BV309	ADGFP	epileptic	14 (2.8)	1	21	74	Minimal HS, P/E

* Animal IDs were numbered sequentially and preceded by the last-name initials of the surgical team. AAVs injected included CSRH1S, a self-complementary AAV containing mCherry driven by the human 0.5 kb synapsin promoter described previously [16], and SmonCeiT, a single-stranded AAV that expresses both mCherry and TREK-M under control of the synapsin promoter and doxycycline [32]. All animals in experiment 1 were treated with doxycycline (AIN-93M chow plus 100 ppm doxycycline, Research Diets, New Brunswick, NJ, USA). Seizure status column includes monitoring conditions of the control animals, where “cage” refers to animals that were left in their home cage and not monitored, while “video” refers to animals housed in clear cages and video-monitored. The following animals were excluded from the results presented: BV311, BV302, and BV305A. BV311 was not injected with pilocarpine but developed spontaneous seizures. Spontaneous seizures in Sprague-Dawley rats have been reported previously [39]. BV311 showed similar GFP signals in the DGCs and MF to those in epileptic rats, thereby excluding off-target effects of pilocarpine. Atrophy was estimated by measuring the size of the dentate plus CA fields and then normalized to the average size in controls. The size of epileptic brains averaged 70% of control. BV302’s and BV305A’s brains were shrunken by greater than 50%, and autofluorescence prevented accurate cell counting; therefore, they were excluded. “Seizures” (sz/day) column shows the total number of convulsive seizures observed in video recordings and their frequency (average recording was 10 days; nd, not determined). We only observed a single seizure in BV304, which occurred before AAV injection. BV307 had 63 seizures the day before perfusion. “Days after last seizure” column shows the number of days after the last observed seizure that the animal was perfused. “Days after injection” column shows the number of days after AAV injection that the animal was perfused. “DGC/slice” refers to the average number of GFP+ DGCs observed. Abbreviations in “Comments” column: GCD, granule cell dispersion; EC for GFP+ neurons in layer V/VI entorhinal cortex; EPhys, electrophysiology; HS, hippocampal sclerosis; OML, outer-molecular-layer projections; P/E, GFP+ neurons in peri- and ectorhinal cortices; SGC, semilunar granule cells.

### 2.6. Measuring the Time Course of Activation of EpiPro after a Single Seizure

**Figure 6:**
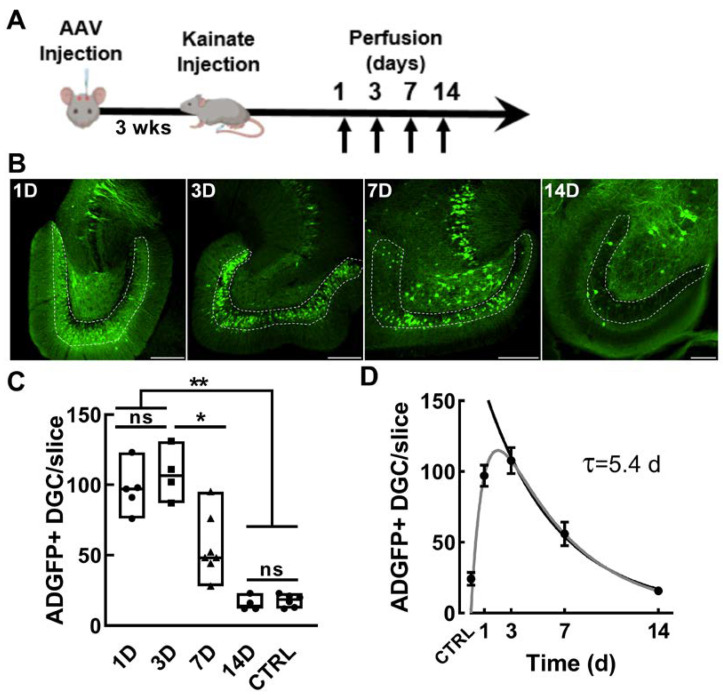
ADGFP activation after a single kainate injection in mice. (**A**), Timeline of experimental procedure. Mice were injected with AAV and then later IP injected with escalating doses of kainate until a motor seizure was elicited (10 ± 5 ± 2.5 mg/kg). Animals were perfused either 1, 3, 7, or 14 days after the KA-induced seizure. (**B**), Representative 10× images from each group of mice (labeled in white, scale bar represents 100 μm). (**C**), The average counts of GFP-labeled dentate granule cells (DGCs) are shown between groups 1D (*n* = 5), 3D (*n* = 3), 7D (*n* = 6), 14D (*n* = 4), and naïve (*n* = 6). Symbols represent each hemisphere that received AAV-scADGFP. Signal in DGCs was at its highest 3 days after a single seizure and then subsequently decreased. By 14 days after a seizure, the signal in the dentate granule cells was equivalent to that in control mice injected with scADGFP. Statistical analysis was carried out with a Brown–Forsythe and Welch ANOVA test followed by Dunnett’s T3 multiple-comparison test. Significance is indicated by asterisks as follows: *, *p* < 0.05; **, *p* < 0.01. There was no significant (ns) difference between 14D and controls (CTRL). (**D**), Average data for each time point (mean ± sem) were first fit with double-exponential equation (gray line, synthesis rate 1 D, decay rate 5.5 D, r^2^ = 0.71), and then the decay phase was fit with a single exponential (black line, τ = 5.4 D; 95% CI 3.6–6.6).

Having established that EpiPro activity is upregulated in dentate granule cells in epileptic animals, we next sought to determine the time course of activation. For these experiments, we used the chemoconvulsant kainic acid (KA), which, when delivered at low doses, can trigger discrete limbic seizures with tonic–clonic motor convulsions [40]. We injected AAV-scADGFP into the dentate gyrus, waited three weeks for expression, and then triggered seizures with an IP injection of 10 mg/kg KA in escalating doses (Figure 6A, Table 2). Mice were then perfused at various time points, and GFP+ cells were counted. Similar to the results in chronic epileptic rats, GFP+ dentate granule cells were low in control mice but substantially higher after a KA-induced seizure (Figure 6B,C). To estimate the time course, we fit the average results to a double exponential curve (Figure 6D). These results indicate that EpiPro is activated rapidly by seizures and then decays slowly, reaching baseline values in 14 days.

### 2.7. Experiment Measuring EpiPro Activity after Mice Stop Having Chronic Seizures

**Figure 7:**
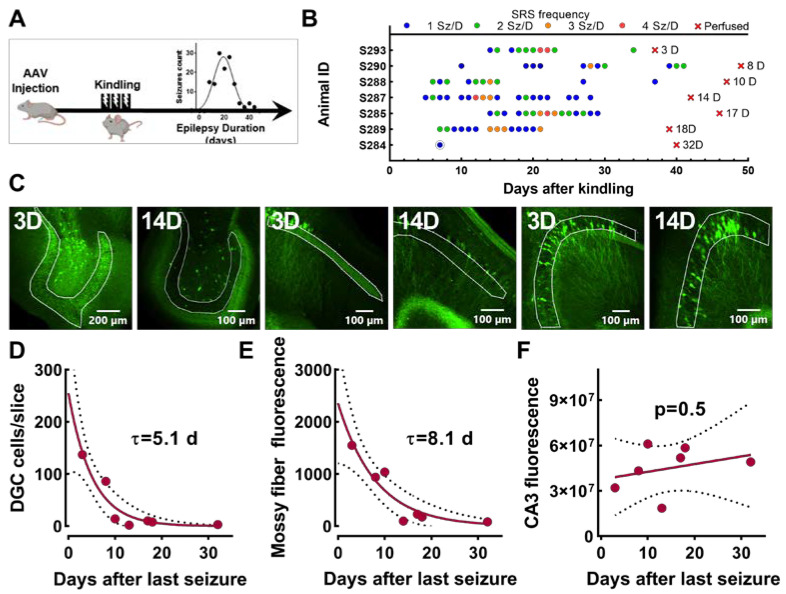
GFP decay in VGAT-Cre mice after seizures end. (**A**), Timeline of experimental procedure. Mice were injected with scADGFP and then later electrically kindled. (**B**), Raster plot of the spontaneous seizures from 7 mice plotted versus time in days from kindling. The color of each dot corresponds to the number of seizures per day, with blue being the least (1 sz/day) and red being the most (4 sz/day). All electrographic seizures were accompanied by a tonic–clonic motor component (average BSS 4.5). The red x’s indicate how many days after the last seizure mice were perfused. (**C**), Images (10×) of dentate granule cells (DGCs), DGC mossy fiber axons (MF), and CA3, respectively, demarcated by white regions of interest (ROIs). Images from either day 3 or 14 after the last seizure. Plots showing the decay of the GFP signal as a function of time after last seizure in DGCs (**D**) and their mossy fiber axons innervating CA3 (**E**). Both data sets were fit with a one-phase exponential decay equation. (**D**–**F**), Fits are displayed as solid curves, and dotted lines represent 95% confidence intervals (CIs): DGC decay, τ = 5.1 (95% CI range 3.1 to 8.3); mossy fiber decay, τ = 8.1 (95% CI range 5.4 to 13). (**F**), Plot of GFP fluorescence in CA3 cell bodies relative to the number of days following the mice’s last seizures. Linear regression indicates the fit was not significantly different from zero (*p* = 0.5).

We have previously shown that electrical kindling of VGAT-Cre mice is sufficient to cause epilepsy [25]. As shown in Figure 7, the seizure frequency peaks 3 weeks after seizures start and approaches zero after 5 weeks. This transient epileptic phenotype allowed us to test whether EpiPro is simply activated by seizures or remains active in between seizures. As detailed in Figure 7A and Table 2, mice were injected with scADGFP, electrically kindled, and video/EEG-monitored. Figure 7B shows the seizure diary for all seven mice that developed spontaneous seizures, as well as how long after their last seizure they were perfused for histology. We then measured GFP+ DGCs and the GFP signal in their mossy fiber axons (Figure 7C–E). These results were fit to a single exponential curve to estimate the time course of decay. Notably, the decay was much slower in axons than in cell bodies. In contrast, the analysis of GFP+ CA3 neurons did not show a significant decay in EpiPro expression in mice (Figure 7F).

## 3. Discussion

This study arose from the following question: how does one target a gene therapy specifically to epileptic neurons? As a first step, we developed a synthetic promoter composed of highly conserved transcription response elements that are upregulated in human epileptic brain tissue [7]. The design of an “epilepsy promoter” (EpiPro) was also guided by an extensive knowledge base on the mechanisms regulating activity-dependent genes [41]. Increased intracellular calcium in neurons plays a key role in the acute and chronic phases of epilepsy [42], while the upregulation of cAMP Response Element-Binding Protein has been demonstrated in human epileptic tissue [7,8]. Therefore, we designed EpiPro to be regulated by calcium and cAMP by adding NFAT, CaRE, and CREB response elements. Notably, Ca/CaM-regulated adenylyl cyclase is strongly expressed in the hippocampus [43]. A distinguishing feature that separates EpiPro from other activity-dependent promoters, such as RAM [6], is that EpiPro is a collection of response elements upregulated in epileptic neurons, each of which is capable of driving gene expression on its own [6]. This approach was taken to increase the targeting of different neuronal populations. Future studies could refine these approaches by only using the response elements that are active in specific subsets of hippocampal neurons.

### 3.1. Validation of the Activity Dependence of EpiPro

Using protocols that reliably increase the firing of cultured neurons (4-aminopyridine plus bicuculline [9]), we found that GFP expression from EpiPro could be upregulated over 100-fold. We used this assay to compare two versions of EpiPro: the core promoter (EPRGFP) and one flanked by regulatory elements from the *Arc* gene promoter (ADGFP, [12,19]). The Arc-flanked EpiPro displayed a 5-fold higher GFP signal, so it was selected for this study. In contrast, commonly used neuronal promoters such as synapsin [44] and Camk2a [45] show minimal activity dependence (Supplementary Figure S2 compares AAV-GFP expression in naive to epileptic mice; synapsin 1.5-fold, *n* = 10 mice; Camk2a, 0.9-fold, *n* = 4 mice).

AAV-mediated expression of TREK-M has been shown to reduce neuronal firing, the duration of SE, and associated neuronal death [30]. As hypothesized, co-expression of TREK-M reduced scADGFP expression both in cultured neurons and in vivo after pilocarpine-induced SE. Electrophysiological studies confirmed these findings: subicular neurons that expressed scADGFP were capable of firing action potentials at higher frequencies than neighboring unlabeled cells. Previous studies on epileptic brains have found increases in the intrinsic firing of neurons throughout the hippocampal circuit (reviewed in [46]). Therefore, EpiPro should prove useful in targeting highly active neurons.

### 3.2. EpiPro as a Biomarker for the Breakdown of the Dentate Gate

Information flow through the hippocampal trisynaptic circuit is heavily filtered at the dentate by high GABAergic tone, resulting in the sparse firing of DGCs. Considerable evidence supports the hypothesis that the breakdown of this filtering function is a critical event in epileptogenesis (reviewed in [22,23,47]). Our findings support this hypothesis, as the most striking and consistent characteristic of EpiPro was its strong labeling of dentate granule cells in epileptic brains versus controls. To exclude the possibility that this result was due to differences in AAV injection or increased AAV uptake by epileptic neurons [48], we developed an “activity index” based on immunohistochemical amplification. We reasoned that EpiPro drove some level of GFP expression in all neurons transduced with AAV, allowing for detection using either native fluorescence when expression was high or anti-GFP immunofluorescence when expression was low. Accordingly, counts of anti-GFP-labeled DGCs exceeded those of native GFP by a factor of 10 in control animals but were closer to 2 in epileptic animals (activity indexes 10% and 50%, respectively). The fluorescent intensity of the GFP signal was also significantly increased: in control animals, the GFP signal was low, with 25% of the neurons being below the threshold used (2-fold above background), while in epileptic animals, GFP expression was increased 2-fold.

### 3.3. Exploration of Epileptic Circuits

The selectivity of EpiPro for chronically active neurons enabled its use as a biomarker in visualizing how neuronal activity progresses from the dentate through the trisynaptic circuit and projects out to thalamic and cortical structures. From the dentate, we characterized DGC projections to CA3 via the mossy fiber tract. DGC projections were translamellar, with DGCs projecting toward the temporal pole [35]. Epileptic animals showed increases in the number of GFP+ neurons and their GFP expression all along the trisynaptic circuit, including CA3, CA1, and the subiculum.

EpiPro drove strong GFP expression in the subiculum and the fornix, and this was significantly increased in epileptic animals (Figure 5). The patch clamping of subicular neurons indicated that EpiPro-expressing GFP+ neurons can fire at higher frequencies than unlabeled neurons (Figure 2). Thus, the increase in EpiPro-driven GFP expression in epileptic neurons is likely due to the increased firing of subicular neurons in epileptic animals [33]. We consistently observed native GFP fluorescence in a variety of thalamic and cortical areas in epileptic animals, including the prefrontal cortex, lateral septum, anterodorsal nuclei, reunions nucleus, nucleus accumbens, and entorhinal cortex layer V. Anti-GFP staining was generally necessary to visualize these projections in control animals, emphasizing the striking increase in hippocampal output associated with TLE. Some of these brain regions only receive hippocampal inputs from subicular neurons (e.g., anterior nuclear group of the thalamus), while most also receive inputs from CA3 and CA1 neurons (e.g., nucleus accumbens and prefrontal cortex; see ref. [36] for complete wiring diagram). The hypothesis that targeting these cortical projections with EpiPro-driven gene therapy may reduce seizures in TLE patients is supported by the clinical efficacy of deep brain stimulation of the anterior thalamus [49].

### 3.4. Activation and Deactivation of EpiPro Promoter Activity

The EpiPro promoter was engineered to contain response elements to transcription factors that are activated in chronic epileptic tissue from human patients [7,8]. In contrast, recent animal studies have used the cFos promoter, an immediate early gene [5] that turns on and off rapidly. The kinetics of cFos expression after spontaneous seizures were studied by Peng and Houser using immunocytochemistry, finding strong expression after 1 h and returning to baseline expression after 2 h [50]. Therefore, a gene therapy based on the cFos promoter would activate after a seizure and provide short-term anti-seizure efficacy afterward. EpiPro was designed with long-term post-seizure activation in mind, which would theoretically provide more robust protection against seizures. It was thus necessary to determine the precise kinetics of EpiPro to assess its utility to drive gene therapies. Our studies used GFP fluorescence as a readout, focusing on both the kinetics after an acute chemoconvulsant seizure and the decay kinetics after spontaneous seizures. To minimize the effects of slow AAV expression, we waited over two weeks after injection to assess kinetics [51]. Our studies using kainic acid to trigger discrete seizures found that EpiPro rapidly led to robust GFP expression after one day. The decay of GFP fluorescence occurred much slower, taking nearly 14 days to return to control levels. A similar time course of decay was observed after spontaneous seizures. It is important to note that these slow kinetics are not due to the slow degradation of GFP, as it has a half-life of just 26 h [20]. A caveat to our chronic epilepsy studies is that kindled VGAT-Cre mice are only epileptic for a couple of weeks. While this was key to our time-course study, we cannot differentiate between decay kinetics after epilepsy ends and kinetics between sporadic seizures, as seen in some patients. An important attribute of EpiPro is its inherent basal activity in hippocampal principal cells; the EpiPro-driven expression of GFP occurred in a fraction of CA3, CA2, CA1, and subicular neurons. In contrast, EpiPro showed only low to non-detectable expression in GABAergic neurons that express either calretinin or parvalbumin (Supplementary Figure S1).

### 3.5. Potential Utility of EpiPro as a Gene Therapy Driver

Taken together, these data indicate that EpiPro should be useful to drive anti-seizure gene therapies, providing basal expression long after a seizure and increased expression that lasts for weeks after a seizure. For example, if EpiPro drives the expression of an ion channel that promotes hyperpolarization, this could work to restore the balance of excitation and inhibition that normally prevents the hyperactivity of vulnerable neurons in TLE, particularly DGCs. However, because EpiPro has basal expression in projection neurons without seizures, care would need to be taken to avoid silencing these neurons completely, which could lead to unwanted cognitive side effects.

## 4. Materials and Methods

### 4.1. Experimental Design

Studies were performed using male Sprague-Dawley rats (200 gm, CD1 strain, Charles River Laboratories, Wilmington, MA, USA) that were singly housed but otherwise maintained under standard feeding and light/dark conditions. Chronic epileptic rats were generated using lithium/pilocarpine, as described previously [30]. We present results from control animals and three experiments on epileptic animals. Experiment 1 used chronic epileptic rats in which a second bout of pilocarpine-induced SE was used to determine the maximal extent of EpiPro activation (Figure 2). Experiment 2 used rats injected with AAV before Li/pilocarpine treatment. Experiment 3 used chronic epileptic rats that were injected with AAV after they had been observed to have spontaneous seizures. Age-matched controls (controls) were injected with the same AAV constructs. Controls included animals that were either maintained in standard cage conditions or video-monitored in Plexiglas cages. After four weeks, animals were perfused, and brains were sliced. Key details of animals used in this study are presented in Table 1. All experiments were performed in accordance with Association for Assessment of Laboratory Animal Care policies. The protocol was approved by the University of Virginia Animal Care and Use Committee.

### 4.2. Cloning of the AAV Targeting Vectors

The core EpiPro promoter and flanking Arc elements were synthesized by Genewiz (Burlington, MA, USA). They were assembled in our self-complementary AAV targeting vector, described previously [30]. A GFP containing the ER-trafficking motif FCYENE [18,29] was codon-optimized and synthesized by Genewiz (GFP-ERX). The AAV encoding TREK-M was a 1st-generation gene therapy activated by doxycycline (Dox-ON; see [32] for details). Its design is similar to the original Dox-OFF AAV [52]. Briefly, it uses the 0.5 kb human synapsin promoter to drive expression of a reverse tetracycline transcriptional activator (rtTA; [28]), which then regulates the expression of both mCherry and TREK-M, a modified leak K+ channel described previously [30]. Both AAVs contain the SV40 early polyadenylation sequence. The nucleotide sequences of the AAV targeting vectors from 5′-ITR to 3′-ITR were confirmed by sequencing (Genewiz). The final constructs were packaged in AAVrh10 particles by either the University of Pennsylvania Vector Core (Philadelphia, PA, USA) or the Horae Gene Therapy Center (Worcester, MA, USA). The titers were as follows (vector genomes/mL): scADGFP, 3.14 × 10^13^; SmonCeiT, 0.5 × 10^13^; and CSRH1S, 1 × 10^13^. Two distinct batches of scADGFP were used.

### 4.3. Lithium/Pilocarpine Treatment and Seizure Monitoring

Animals were administered 50 mg/kg of pilocarpine intraperitoneally (IP) to induce SE. Pretreatments before Li/pilocarpine injection included both lithium chloride (3 mmol/kg; 24 h before) and methyl-scopolamine (1 mg/kg; 40 min before). All animals injected with Li/pilocarpine developed tonic–clonic seizures. Motor convulsions were terminated by diazepam injection 2 h later (10 mg/kg). Four weeks following Li/pilocarpine treatment, control and pilocarpine-treated animals were video-monitored to detect spontaneous motor seizures (24 h/d, /7 d/wk). Animals were housed in clear Plexiglas cages with wire bottoms during monitoring. 

### 4.4. Stereotaxic AAV Injection

Animals were anesthetized with isoflurane and monitored for depth of anesthesia throughout the procedure. Four holes were drilled into the skull for AAV injection on both hemispheres at the following coordinates (distance from bregma): 4.5 mm lateral and 6.0 mm posterior to target the hilus; 5.0 mm lateral and 7.1 mm posterior to target the subiculum [38]. At each hole, 3 μL of virus was delivered, starting at a depth of −7.6 mm and ending at −4.1 mm. AAV was infused by convective delivery using a Nanofil syringe (33-gauge needle) continuously driven by a microsyringe pump controller (Micro4, World Precision Instruments, Sarasota, FL, USA). After injection, the skin was sutured, and 0.1 mL ketoprofen was IP injected.

### 4.5. Perfusion and Brain Slicing

Animals were anesthetized with 0.5 mL of pentobarbital and then perfused with 100 mL of saline, followed by 400 mL of freshly prepared 4% paraformaldehyde (PFA). Brains were post-fixed further in PFA for 24–48 h (at 4 °C). A small hole was made in the rostral cortex of the right hemisphere to orient the slices. Brains were embedded in agarose and cut into 40 μm horizontal slices with a Leica VT 1000S vibratome (Leica Biosystems, Deer Park, IL, USA). To allow comparisons at a particular depth, brain slices were transferred in order to a multi-well plate and then stored at −20 °C in cryoprotectant (30% glycerol, 30% ethylene glycol, 0.2 M phosphate buffer).

### 4.6. Imaging and Analysis

Stereological techniques were used. Imaging was performed on 10 evenly spaced brain slices from each animal, corresponding to −4 to −8.5 mm below bregma for rats and −2 to −5 mm for mice. Slices were cleared using 2,2’-thiodiethanol [53] and then mounted on slides with Fluoromount-G plus DAPI (Southern Biotech, Birmingham, AL, USA). Each slide contained two slices covered with a single coverslip (#1 glass). Depth was localized for each brain using the anterior commissures (e.g., −7.1 mm from bregma for rats [38]). Subfields imaged included the dentate gyrus, hilus, CA fields, subiculum, parasubiculum, entorhinal cortex, fornix, anterior thalamic nuclei, septum, prefrontal cortex, and nucleus accumbens. To prevent double-counting of neurons, each subfield was included in a single image when possible. Images were acquired using an Olympus IX 81 microscope equipped with a Nipkow spinning-disc confocal unit. Imaging and analysis used cellSens software (Olympus, Tokyo, Japan, RRID:SCR_016238). Cell counting was performed on images acquired at 10× magnification. Colocalization was performed using confocal Z-stack images acquired at 20× magnification (Supplemental Figure S1). Three fluorescent cubes were utilized: (1) for native GFP signal, ex. 470/40 nm, em. 525/50 nm; for red Alexa-conjugated secondary antibodies, ex. 562/40 nm, em. 624/40 nm; and for blue DAPI nuclei staining, ex. 350/50 nm, em. 460/60 nm.

Regions of interest (ROIs) were drawn around each subfield to be analyzed, and neurons were counted using the adaptive thresholding tool in cellSens. This algorithm measures the local background around an object, allowing for its detection even when autofluorescence is high and variable. This intensity adaptation was fixed to a local value of 20. The minimum adaptive threshold for object identification was 150. Only cell bodies with a diameter greater than 25 μm were counted. All analysis was performed by investigators blinded to each animal’s experimental condition. To confirm the accuracy and consistency of this cell-counting procedure, two sets of slices from 5 animals were counted by different investigators, with no significant differences in the cells counted (101 + 17%).

The location of subfields followed the standard definition illustrated in the horizontal sections of the rat brain atlas [38]. Boundaries for cell count analysis were as follows. ROIs for the DGC layer were drawn around the prominent blue DAPI staining of granule cell nuclei (example shown in Figure 6B). The hilus was defined as the area inside the DG, excluding any pyramidal cells from the descending CA3 layer. In preliminary studies, the location of CA2 was visualized by RGS14 staining [34]. Subsequent measurements defined CA2 using the tip of the stratum lucidum. The subiculum was defined starting from the end of the CA1 body layer and ending at the presubiculum.

Projections in the inner molecular layer (IML), mossy fiber tract (MF), and fornix were analyzed to determine their GFP signal intensity. The IML, which is mainly innervated by mossy cells [54], was defined as the innermost third of the molecular layer adjacent to the dentate granule cell layer. The MF, which is innervated by dentate granule cell axons [54], was visible as a band adjacent to CA3. The fornix, which carries the main output from the hippocampus [36], was measured in cross-sections of the descending limb. ROIs were drawn around representative portions of the projection, and an adaptive threshold of 25 was used to measure average signal intensity. Background signal was measured using a manual threshold of 0 in adjacent tissue and then subtracted from average intensity measured in the projection. The background signals in the middle molecular layer (MML) were used to correct both the IML intensity and to develop minimum criteria for signal detection. Thus, active neurons were defined as those with native GFP signal intensity that was 2× greater than the average background measurement for that animal.

We devised a method to determine whether the lack of GFP fluorescence was due to either the lack of AAV transduction or low neuronal activity. This method relies on antibody amplification of the green GFP signal using a chicken anti-GFP primary antibody and a red secondary antibody (goat anti-chicken antibody conjugated to Alexa-555). Under our experimental conditions, the anti-GFP signal was 52-fold higher than the native GFP signal, allowing the detection and counting of neurons with very low GFP expression. The ratio of neurons counted with native GFP to those counted after anti-GFP staining thus defines the “activity index” for each region analyzed.

### 4.7. Immunohistochemistry

Slices were washed in phosphate-buffered saline (PBS) three times for 15 min and then blocked for 1 h in 0.5 mL of blocking solution (100 mM PBS, 0.005% bovine serum albumin, 0.4% Triton X-100, and 10% donkey serum). Slices were washed again and immersed in the primary antibody solution (10 mM PBS, 1% donkey serum, 0.1% sodium azide, 0.4% Triton X-100, and primary antibody) for 48 h at room temperature with continuous agitation. After a third washing step, slices were immersed in the secondary antibody solution (10 mM PBS, 1% serum, 0.1% sodium azide, 0.4% Triton X-100, and secondary antibody) and agitated continuously for 24 h. Slices were washed a final time with PBS, mounted onto 0.1% gelatin-subbed slides, embedded in DAPI-Fluoromount (Southern Biotech), and coverslipped. Details of the primary antibodies used are as follows: anti-GFP, chicken polyclonal (Aves, Davis, CA, USA, RRID:AB_10000240, 1:1000); anti-RGS14 monoclonal antibody (Neuromab, Davis, CA, USA, RRID:AB_10698026). Details of the secondary antibodies used are as follows (all diluted 1:1000): goat anti-chicken Alexa Fluor^®^ 555 (ThermoFisher Scientific, Waltham, MA, USA, A-21437, RRID:AB_2535858); goat anti-mouse Alexa Fluor^®^ 568 (Thermo Fisher Scientific, #A-21134, RRID:AB_2535773).

### 4.8. Brain Slice Electrophysiology

Electrophysiological recordings in current-clamp mode were performed using horizontal brain slices (350 μm thick) as previously described [33]. Subiculum neurons were identified by infra-red video microscopy using a ZEISS Axioscope microscope (ZEISS Group, Oberkochen, Germany). Action potentials were evoked with a series of current injection steps from −20 pA to +470 pA in 10 pA steps for 300 ms at 5 sec inter-pulse intervals. To standardize our measurements, the resting membrane potential (RMP) was recorded and then maintained at −65 mV by injection of DC current.

### 4.9. Statistical Analysis

The experimental design used separate injections of AAV into the left and right hemispheres of the brain, which resulted in differences in the extent of labeling. Therefore, we included data from both hemispheres in the statistical analysis, such that *n* represents the number of hemispheres, except as noted. The distribution of data points was evaluated using the D’Angostino and Pearson normality tests. If data from control and epileptic animals both had normal distributions, then statistical significance was determined using Student’s *t*-test. If the distribution of data from either set was not normal, then statistical significance was determined using the Mann–Whitney test. Cumulative distributions of GFP fluorescence in neurons were compared using the Kolmogorov–Smirnov test. Data with repeated measures were analyzed by 2-way ANOVA and Sidak’s multiple-comparison test. All calculations of *p* value used two-tailed tests. Data reported in the text are mean ± SEM. All statistical analysis was performed using Prism software (GraphPad Software, Boston, MA, USA, version 8.4; RRID:SCR_002798).

## 5. Conclusions

The EpiPro promoter was designed with the goal of selectively targeting hyperactive neurons of the hippocampus to drive a gene therapy exclusively in those neurons, with the ultimate goal of stopping seizures and minimizing off-target effects. After establishing EpiPro as an activity-modulated promoter, we observed that epileptic animals have consistently strong expression of EpiPro in DGCs, while control animals have little to none. This result provides direct in vivo support for the breakdown of the dentate gate theory in TLE and supports the future use of EpiPro as a gene therapy. Tracing GFP+ projections also allowed us to illuminate the circuitry underlying TLE, which facilitates the spread of seizures throughout the brain. EpiPro showed strong basal expression in subsets of hippocampal projection neurons in control animals, implying that an EpiPro-driven gene therapy could provide long-lasting anti-seizure control during seizure-free periods. In contrast, a cFos promoter would only be activated immediately following a seizure, providing a much more limited window for reducing the activity of these neurons. The selectivity of EpiPro for hyperactive neurons, its rapid induction by seizure activity, and the maintenance of expression for days following seizures all establish this promoter as a viable candidate for driving gene therapies in focal epilepsies.

## Figures and Tables

**Table 2:** Key features of mouse subjects *.

Animal ID	AAV Injected	Seizure Status	Sz/Day	Perf. after Last Sz	Perf. after inj.	DGC/Slice
**Controls**						
G054	ADGFP	control	na	na	18	7
G055	ADGFP	control	na	na	19	15
G086	ADGFP	control	na	na	69	23
G092	ADGFP	control	na	na	64	6
S179	ADGFP + CSRH1S	control	na	na	34	25
S180	ADGFP + CSRH1S	control	na	na	34	41
S181	ADGFP + CSRH1S	control	na	na	33	31
S183	ADGFP + CSRH1S	control	na	na	33	25
S184	ADGFP + CSRH1S	control	na	na	32	45
**Experiment 1: Chronic epilepsy triggered by electrical kindling**						
G051	ADGFP	K + SRS	2.3	1	54	73
G053	ADGFP	K + SRS	2.6	1	43	30
G084	ADGFP	K + SRS	1.1	0	71	65
G091	ADGFP	K + SRS	0.2	3	59	109
G093	ADGFP	K + SRS	nd	2	56	55
G095	ADGFP	K + SRS	3.4	1	55	64
**Experiment 2: Time course after single kainate-induced seizure**						
S136	ADGFP + CSRH1S	10 mg KA	na	1	22	110
S138	ADGFP + CSRH1S	15 mg KA	na	1	21	94
S143	ADGFP + CSRH1S	10 mg KA	na	1	18	76
S122	ADGFP + CSRH1S	17 mg KA	na	3	66	87
S123	ADGFP + CSRH1S	10 mg KA	na	3	65	116
S127	ADGFP + CSRH1S	15 mg KA	na	3	62	111
S137	ADGFP + CSRH1S	10 mg KA	na	7	27	85
S139	ADGFP + CSRH1S	10 mg KA	na	7	26	50
S140	ADGFP + CSRH1S	10 mg KA	na	7	26	46
S141	ADGFP + CSRH1S	10 mg KA	na	7	25	28
S142	ADGFP + CSRH1S	10 mg KA	na	14	31	14
S144	ADGFP + CSRH1S	10 mg KA	na	14	31	23
S145	ADGFP + CSRH1S	15 mg KA	na	14	28	12
**Experiment 3: Time course after epilepsy ends**						
S293	ADGFP + CSRH1S	K + SRS	1.10	3	42	137
S290	ADGFP + CSRH1S	K + SRS	0.52	8	54	86
S288	ADGFP + CSRH1S	K + SRS	0.52	10	54	14
S287	ADGFP + CSRH1S	K + SRS	1.17	13	47	2
S285	ADGFP + CSRH1S	K + SRS	1.87	17	54	10
S289	ADGFP + CSRH1S	K + SRS	1.79	18	47	8
S284	ADGFP + CSRH1S	K + SRS	na	32	47	3
S291	ADGFP + CSRH1S	K + SRS	na	na	55	9
S292	ADGFP + CSRH1S	K + No SRS	na	na	44	6

* Seizure status column includes the following: AAV-only controls; mice that were electrically kindled (K) and developed spontaneous recurring seizures (K + SRS; [25]); kindled mice that did not develop epilepsy (K + No SRS); and mice that received a dose of kainic acid that triggered a single motor seizure (dose indicated). Seizure frequency was determined using 24/7 video/EEG monitoring during a 2-week period after the 2nd spontaneous seizure. Also shown is the time between injection and perfusion and the average DGC count in each animal.

## Data Availability

The data presented in this study are available in this article and upon request. The DNA sequence of scADGFP will be available on Addgene upon publication (ID# 114434).

## Supplementary Materials

The following supporting information can be downloaded at https://www.mdpi.com/article/10.3390/ijms241914467/s1. References [55,56,57,58,59] are cited in the supplementary materials.
