# Supplementary material for: EpiPro, a Novel, Synthetic, Activity-Regulated Promoter That Targets Hyperactive Neurons in Epilepsy for Gene Therapy Applications
Source: Int J Mol Sci. 2023 Sep 23;24(19):14467. doi: 10.3390/ijms241914467 (PMC10572392; doi:10.3390/ijms241914467)
Supplement: Supplementary file 1 [file ijms-24-14467-s001.zip › ijms-2590618-supplementary.pdf]

Supplementary Figure 1

Materials and Methods

Expression of scADGFP in neuronal subtypes in the dentate hilus

Colocalization of GFP+ cells with staining for glutamatergic (GluR2/3) and GABAergic neuron markers (calretinin, and parvalbumin) was performed using cellSens software (Olympus, Tokyo, Japan). Brain slices were stained with either: 1) rabbit anti-GluR2/3 antibody (MilliporeSigma, Burlington, MA, USA, RRID:AB_90710) and donkey anti-rabbit Alexa Fluor 594, Jackson ImmunoResearch Labs, West Grove, PA USA, RRID:AB_2340621); 2) goat anti-calretinin antibody (MilliporeSigma, RRID:AB_2174013) and goat anti-mouse Alexa Fluor 549 RRID:AB_2340435); and 3) mouse monoclonal anti-parvalbumin antibody (MilliporeSigma, clone PARV-19, RRID:AB_2857970) and goat anti-mouse Alexa Fluor 568 (Jackson ImmunoResearch). Z-stack images of the dentate hilus were taken with a 20x objective. GluR2/3+ and GFP+ cells were counted for each image, and the counts of all GluR2/3 positive, GFP positive, and colocalized cells were recorded. Most GluR2/3 positive cells observed had similar signal intensity. However, the anti-calretinin and anti-parvalbumin) fluorescence in slices stained for those markers was slightly more variable. To ensure that only cells with above-background fluorescence were counted as calretinin+ or parvalbumin+, a consistent adaptive threshold was identified that reliably identified labeled cells with above-background signal. To analyze each image, the appropriate adaptive threshold was first applied on the red channel, and all cells with above-threshold signal were marked with a point ROI. Then, the GFP adaptive threshold was applied on the green channel, and the count of GFP+ cells was recorded. To count colocalized cells, ROIs were drawn around cells that were both marked with a point ROI (Alexa 594/568+) and counted by the GFP adaptive threshold (GFP+). The count, intensity, and area of all colocalized cells were recorded

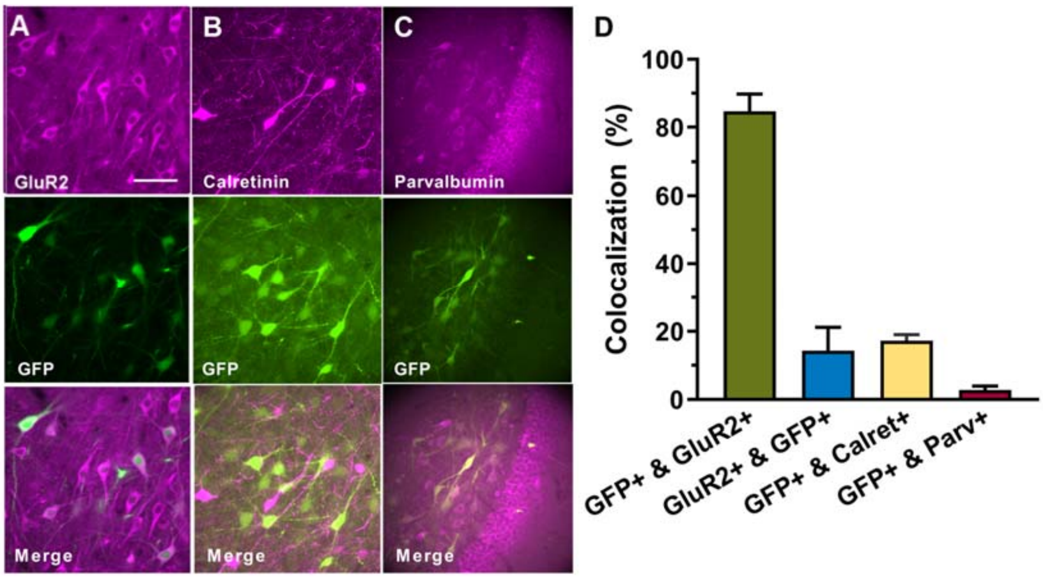

Supplementary Fig. 1 legend: Characterization of ADGFP labeled neurons in the dentate hilus. Representative images of anti-GluR2/3 (**A**), anti-calretinin (**B**), and anti-parvalbumin (**C**) stained slides, the corresponding native GFP image, and the merge. Excitatory neurons express glutamate-sensitive AMPA receptors, and in the dentate hilus, only Mossy cells express GluR2/3 [1]. Slices were stained for the GluR2 subunit of these receptors to determine both the fraction of EpiPro labeled neurons that are GluR2+ (GFP+ & GluR2+), and the fraction of GluR2+ neurons that expressed EpiPro (GluR2+ & GFP+).

Expression of calretinin in Mossy cells is species specific, having been documented in mouse, but not in rat or human [2]. **D,** Extent of colocalization of GFP+ neurons with staining for GluR2/3, calretinin, and parvalbumin. Colocalization of GFP+ neurons in scADGFP naives with GluR2 staining revealed that 85 ± 2% (n=5) of GFP+ neurons are also GluR2 positive. However, only 14 ± 3% (n=5) of the GluR2+ Mossy cells were GFP+. Slices were also stained for calretinin and parvalbumin, which target two distinct interneuron subtypes [3]: 17 ± 2% (n=3) of GFP+ neurons were Calretinin+ and 3 ± 1% (n=5) were Parvalbumin+. N represents the number of animals, where the result for each animal is the average of 4 slices. Scale bar shown in **A** represents 100 μm for all images.

References

1. Volz, F.; Bock, H. H.; Gierthmuehlen, M.; Zentner, J.; Haas, C. A.; Freiman, T. M., Stereologic estimation of hippocampal GluR2/3- and calretinin-immunoreactive hilar neurons (presumptive mossy cells) in two mouse models of temporal lobe epilepsy. *Epilepsia* **2011,** 52, (9), 1579-89. [10.1111/j.1528-1167.2011.03086.x]
2. Seress, L.; Abraham, H.; Czeh, B.; Fuchs, E.; Leranth, C., Calretinin expression in hilar mossy cells of the hippocampal dentate gyrus of nonhuman primates and humans. *Hippocampus* **2008,** 18, (4), 425-34. [10.1002/hipo.20403]
3. Freund, T. F.; Buzsáki, G., Interneurons of the hippocampus. *Hippocampus* **1996,** 6, (4), 347-470. [10.1002/(SICI)1098-1063(1996)6:4<347::AID-HIPO1>3.0.CO;2-I]

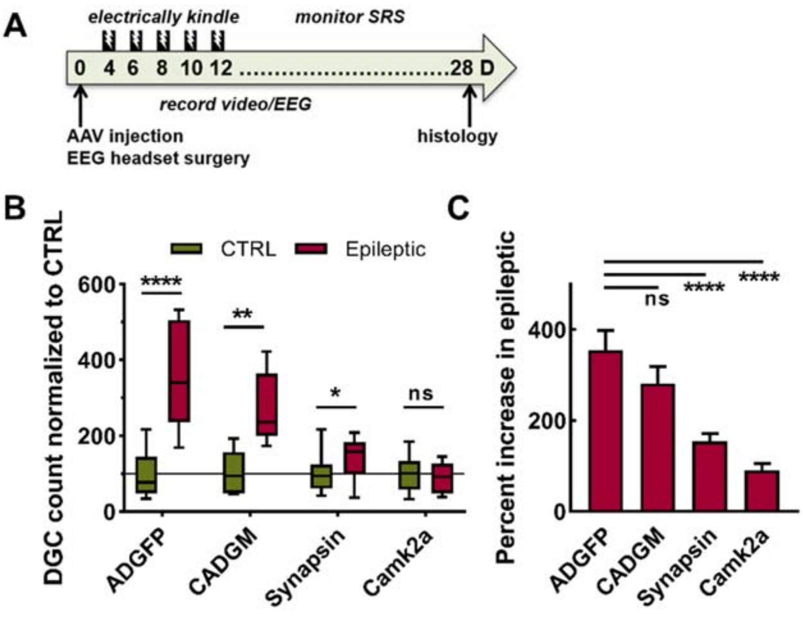

Supplemental Fig. 2 Comparison of the activity dependence of various promoters in dentate granule cells using control and epileptic VGAT-Cre mice. **A.** Experimental plan to test promoter activity in epileptic VGAT-Cre mice. Electrical kindling of VGAT-Cre mice leads to spontaneous recurring seizures (SRS) after a short 10 day latency as detailed in our previous studies [1, 2]. Control mice were only injected with AAV. We compared the following promoters: EpiPro-flanked with Arc elements (scADGFP, Addgene #114434); EpiPro flanked with a 0.9-kb fragment of the mouse Camk2a promoter (unpublished, Camk2a promoter described in [3]); a 0.5-kb fragment of the human synapsin promoter (CSRH1S; [4]), and a 0.4-kb fragment of the mouse Camk2a promoter (Addgene # 105541, a gift from James M. Wilson). These promoters drove the expression of either GFP (ADGFP, CADGM, Camk2a) or mCherry (Synapsin). **B,** Fluorescently-labeled dentate granule cells were counted in 10x images of horizontal brain slices. The values obtained in each mouse was normalized to the average values obtained in control mice. The number of VGAT-Cre mice studied for each promoter was as follows: ADGFP4, n=10, control, n=8, epileptic; CADGM, n=4 control, n-4 epileptic; Synapsin, n=10, control, n=8, epileptic; and Camk2a, n=7, control, n=4, epileptic. Statistical analysis in by two-tailed t-test (****, p<0.0001; **, p<0.01, *, p,0.05, ns, p>0.05). **C**, Results shown in panel B were replotted to highlight the increase in promoter activity in epileptic mice. Statistical analysis by 1-way ANOVA (****, p<0.0001; ns, p>0.05; Prism software (GraphPad Software, Boston, MA, USA, version 8.4; RRID:SCR_002798).

References

1. Straub, J.; Gawda, A.; Ravichandran, P.; McGrew, B.; Nylund, E.; Kang, J.; Burke, C.; Vitko, I.; Scott, M.; Williamson, J.; Joshi, S.; Kapur, J.; Perez-Reyes, E., Characterization of kindled VGAT-Cre mice as a new animal model of temporal lobe epilepsy. *Epilepsia* **2020,** 61, (10), 11. [10.1111/epi.16651]
2. Straub, J.; Vitko, I.; Gaykema, R. P.; Perez-Reyes, E., Preparation and Implantation of Electrodes

for Electrically Kindling VGAT-Cre Mice to Generate a Model for Temporal Lobe Epilepsy. *Journal of Visualized Experiments* **2021,** e62929, (174). [10.3791/62929]
3. Dittgen, T.; Nimmerjahn, A.; Komai, S.; Licznerski, P.; Waters, J.; Margrie, T. W.; Helmchen, F.; Denk, W.; Brecht, M.; Osten, P., Lentivirus-based genetic manipulations of cortical neurons and their optical and electrophysiological monitoring in vivo. *Proc Natl Acad Sci U S A* **2004,** 101, (52), 18206-11. [10.1073/pnas.0407976101]
4. Park, J.; Yu, Y. P.; Zhou, C. Y.; Li, K. W.; Wang, D.; Chang, E.; Kim, D. S.; Vo, B.; Zhang, X.; Gong, N.; Sharp, K.; Steward, O.; Vitko, I.; Perez-Reyes, E.; Eroglu, C.; Barres, B.; Zaucke, F.; Feng, G.; Luo, Z. D., Central Mechanisms Mediating Thrombospondin-4-induced Pain States. *J Biol Chem* **2016,** 291, (25), 13335-48. [10.1074/jbc.M116.723478]
